# Recent Advancements in Optical Harmonic Generation Microscopy: Applications and Perspectives

**DOI:** 10.34133/2021/3973857

**Published:** 2021-01-25

**Authors:** Darian S. James, Paul J. Campagnola

**Affiliations:** Department of Biomedical Engineering, University of Wisconsin-Madison, 1550 Engineering Dr, Madison, WI 53706, USA

## Abstract

Second harmonic generation (SHG) and third harmonic generation (THG) microscopies have emerged as powerful imaging modalities to examine structural properties of a wide range of biological tissues. Although SHG and THG arise from very different contrast mechanisms, the two are complimentary and can often be collected simultaneously using a modified multiphoton microscope. In this review, we discuss the needed instrumentation for these modalities as well as the underlying theoretical principles of SHG and THG in tissue and describe how these can be leveraged to extract unique structural information. We provide an overview of recent advances showing how SHG microscopy has been used to evaluate collagen alterations in the extracellular matrix and how this has been used to advance our knowledge of cancers, fibroses, and the cornea, as well as in tissue engineering applications. Specific examples using polarization-resolved approaches and machine learning algorithms are highlighted. Similarly, we review how THG has enabled developmental biology and skin cancer studies due to its sensitivity to changes in refractive index, which are ubiquitous in all cell and tissue assemblies. Lastly, we offer perspectives and outlooks on future directions of SHG and THG microscopies and present unresolved questions, especially in terms of overall miniaturization and the development of microendoscopy instrumentation.

## 1. Introduction

Multiphoton microscopy (MPM) has revolutionized biological imaging since its modern inception in the early 1990s, where the first breakthroughs utilized two-photon excited fluorescence (TPEF) to probe live cells and tissues [1]. This technology largely solved long-standing problems of confocal microscopy including photobleaching and penetration depth limitations. By the late 1990s, other nonlinear optical methods such as second harmonic generation (SHG) [2], third harmonic generation (THG) [3], and coherent anti-Stokes Raman scattering (CARS) [4] were demonstrated as viable biological imaging tools, with each providing its own unique and often complementary information content. While it is more common to pair SHG and TPEF microscopies given the vast array of engineered fluorescent proteins and autofluorescent extracellular matrix (ECM) proteins (e.g., elastin), combining SHG and THG microscopies also has its advantages. These include increased imaging depths due to longer excitation wavelengths and applicability to a wide range of tissues, even those that do not autofluoresce. For example, the sensitivity of THG to refractive index can give structural context for the SHG collagen contrast. Moreover, there are theoretical commonalities (further explained in Section 3) between the two modalities. Therefore, in this review, we will focus on the fundamental principles and emerging applications of SHG and THG microscopies.

## 2. SHG and THG Historical Origins and Overview of Current Uses

**Figure 1:**
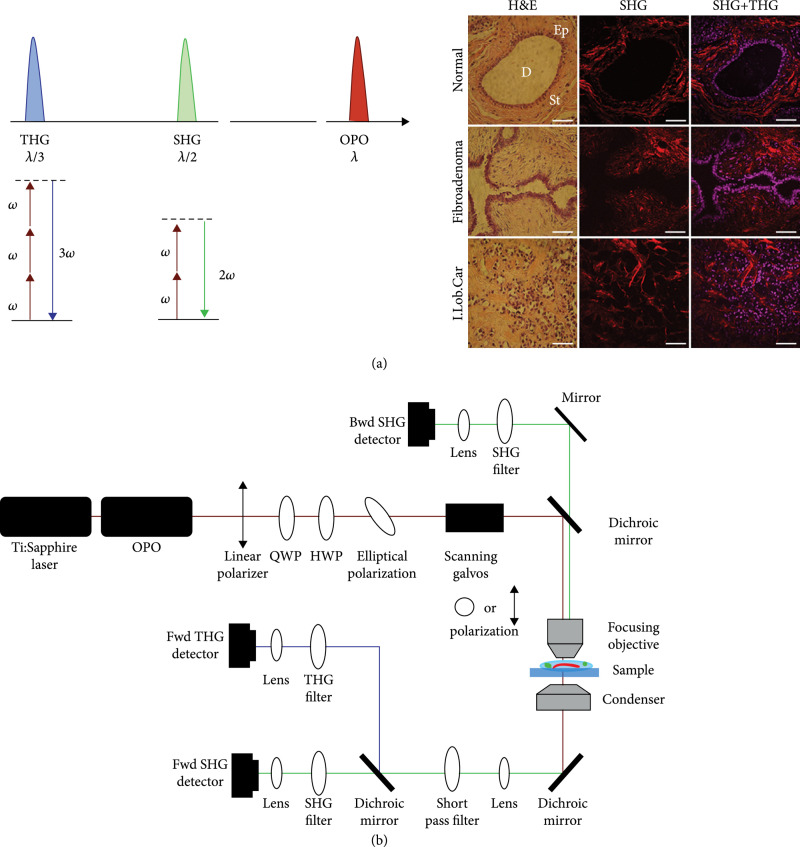
Multimodal nonlinear microscopy. (a) Spectral representation (top) of excitation wavelength and resultant emission wavelengths for THG and SHG. Corresponding Jablonski diagrams (bottom), where dashed lines represent virtual electronic states. Representative H&E, SHG, and SHG+THG images of breast tissues diagnosed as normal (first row), fibroadenoma (second row), and invasive lobular carcinoma (third row). (b) Simple scheme of multimodal microscope beam path for nonlinear imaging. Reproduced from Ref. [14] published under CC BY 3.0, adapted from Ref. [28] published under CC BY 4.0.

SHG is a nonlinear second-order coherent process where two lower energy photons are upconverted, emitting a photon at exactly twice the frequency of the incident excitation source (Figure 1(a)) [5]. Dr. Maria Goeppert-Mayer theoretically predicted SHG (along with two-photon excitation) in her 1931 PhD thesis [6], whereas the first experimental demonstrations were on quartz in 1961 following the development of the ruby laser [7]. While modern SHG biological imaging was reported in the late 1990s [8, 9], it is interesting to note that there were prior spectroscopic and low-resolution microscopy examinations of collagen in 1971 [10] and 1986 [11], respectively.

The initial interest in this contrast mechanism for biological microscopy was probing membrane potential in live cells using voltage-sensitive dyes [12], where it was shown that SHG afforded greater sensitivity than traditional fluorescence methods [5, 8, 13]. However, the larger majority of SHG microscopy has been performed on tissues for structural analysis and we will limit our scope to those applications [2, 14, 15].

SHG microscopy has now emerged as a powerful and widely used tool for high-resolution, high-contrast, three-dimensional imaging of tissues [16]. As will be described in detail in the theory section, SHG contrast requires non-centrosymmetric assemblies on the size scale of $\lambda_{\text{SHG}}$, which is ideal for imaging well-ordered structures such as fibrillar collagen (i.e., Col I, Col II, Col III, and Col V or mixtures thereof). Other structural proteins such as nonfibrillar collagen (i.e., Col IV), laminin, fibronectin, and elastin are transparent by this modality as this criterion is not met. While this may seem a large limitation, collagen is the primary protein component in the extracellular matrix (ECM) of many connective tissues including tendon [17], skin [18], cornea [19], blood vessels, and bone and also in stroma in internal organs such as ovary [20], cervix [21], lung [22], liver [23], and kidney [24]. Indeed, most SHG microscopy applications have focused on probing collagen changes in a wide range of pathologies involving these and other tissues and some of these studies will be reviewed here.

Similar to SHG, THG is a nonlinear coherent process where three photons are upconverted to produce a photon that is triple the frequency of the incident photon (Figure 1(a)). THG was first experimentally found in calcite, gases, and liquids, shortly after the demonstration of SHG [15]. During the late 1990s, high-resolution THG biological microscopy was first demonstrated by the Silberberg, Brakenhoff, and Wilson groups [25, 26]. Unlike SHG, which arises from asymmetries on the size scale of $\lambda_{\text{SHG}}$, THG contrast arises from the 3D volume around interfacial regions with a change of refractive index [27]. Since all cells and tissues have such changes in refractive index, THG can be an effective general imaging tool to map structural distributions in cells and tissues. For example, the sensitivity of THG to cell membranes and other border regions has been successfully utilized to image unstained lipid bodies in cells and whole zebrafish embryos [27]. We note that although THG and SHG arise from very different contrast mechanisms the two modalities are complementary, enabling 3D visualization of different aspects of cells and the ECM (Table 1).

**Table 1:** Comparison of nonlinear optical microscopy techniques. Inspired by Ref. [15].

Method	Primary information	Signal dependence on concentration	Lasers	Contrast source	Emission direction	Applicability	Detection wavelength
SHG	Structural/assembly	Quadratic	Fs Ti:sapph	Fibrillar collagen, actomyosin	F/B tissue dependent	Bulk tissues	Near UV-visible
THG	Structural/assembly	Quadratic	Ti:sapph pump+OPO, Cr:forsterite, custom-built filtered supercontinuum, ytterbium	Change in refractive index	F/B tissue dependent	Interfaces in cells, tissue	Near UV
TPEF	Protein sensing/function/localization	Linear	Fs Ti:sapph	Dye, intrinsic fluorophores	4*π*	Cells, tissues	Visible
CARS	Chemical	Quadratic	Ps pump source (ti:sapp or Nd:YVO_4_)+OPO	Mostly C-H lipids	Primarily forward	Cells, tissues	Visible

## 3. SHG and THG Theory/Photophysics

Given that the contrast from SHG and THG directly arises from their respective physical origins, here we provide an overview of the underlying nonlinear optical theory of each. A material’s response to an applied electric field E can be described using polarization P according to the following relationship:

(1)P=χ1E1+χ2E2+χ3E3+⋯,

where $\chi^{\left(n\right)}$ is the n^th^-order nonlinear susceptibility. The nonlinear effects are achieved at higher order susceptibility (n>1). SHG and other relatively similar nonlinear processes (i.e., sum frequency generation (SFG) and difference frequency generation (DFG)) are governed by $\chi^{\left(2\right)}$ [15]. The third-order susceptibility, $\chi^{\left(3\right)}$, gives rise to THG in addition to two-photon and three-photon absorption, CARS, and stimulated Raman scattering (SRS).

The susceptibility tensor, $\chi^{\left(2\right)}$, is a bulk property and is the quantity measured in an experiment. However, the molecular level property of the nonlinearity, i.e., the first hyperpolarizability, β, forms the basis of the contrast mechanism. This parameter is defined in terms of the permanent dipole moment:

(2) d2=βE2.

**Figure 2:**
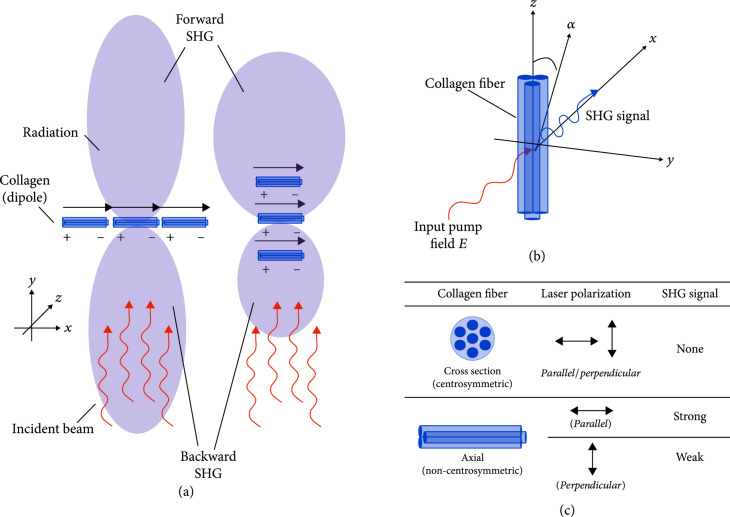
SHG from collagen. (a) Forward and backward SHG emission from radiating dipoles (collagen fibers) oriented parallel and perpendicular to the laser propagation. (b) Excitation and emission of a single collagen fiber. (c) Collagen fiber orientation with respect to laser polarization gives rise to either zero, strong, or weak SHG signal. Reproduced from Ref. [29] published under CC BY 4.0.

The molecular and bulk properties are then related by

(3)χ2=Nsβ,

where $N_{s}$ is the density of molecules and the brackets denote their orientational average. Thus, harmonophores must have a permanent dipole moment, where these must be aligned within the focal volume of the microscope so that $\chi^{\left(2\right)}$ is nonzero (Figure 2). These constraints limit the different proteins that can be visualized with SHG, where the main species are collagen and myosin. In comparison, other matrix proteins do not have either regular molecular structures or assemblies thereof. Additionally, sample orientation with respect to laser polarization gives rise to either zero, strong, or weak SHG signal, where linear polarization preferentially excites specific dipoles (Figure 2(c)) and circular polarization excites all dipoles equally.

While there has been interest in imaging skeletal muscle, the large majority of SHG microscopy has been performed on tissues comprised primarily or partially of type I collagen (or Col I), which is the most abundant protein in the body. It is thus important to elucidate the contrast mechanism in terms of the collagen molecular structure. For example, Schanne-Klein and coworkers used Hyper-Rayleigh Scattering (HRS) measurements to show that the hyperpolarizability, β, arose from coherent amplification of peptide bonds along the length of the molecule [28]. This finding was consistent with our analysis using polarization-resolved measurements, which revealed that the nonvanishing matrix elements governing $\chi^{\left(2\right)}$ can be related to the pitch angle (~50 degrees) of the individual *α*-helices in the collagen molecule (see Section 5.3) [30].

THG is a third-order nonlinear process, which involves a real $\chi^{\left(3\right)}$ susceptibility and is related to the nonlinear refractive coefficient $n_{2}$ by

(4)n2=34ε0n02cReχ3,

where $\varepsilon_{0}$ is the vacuum dielectric constant, $n_{0}$ is the linear refraction coefficient (${n_{0}}^{2}\propto Re\left\{\chi^{\left(1\right)}\right\}$), and Re denotes the real component of a complex value [31]. Thus, THG is sensitive to inhomogeneities such as aqueous medium interfaces and microstructures where $n_{2}$ is highly mismatched [25]. Despite the lower peak power, the THG intensity can be larger using a lower NA objective, as the signal arises from the volume around the region of refractive index difference, rather than the interfacial region itself. Consequently, this modality does not provide strong contrast for bulk volumes in tissue with similar refractive index, e.g., bands of collagen that still provide the same asymmetry needed for SHG.

## 4. Microscope Setup/Instrumentation

Here, we provide the salient equipment aspects that are specific to SHG and THG microscopes (Figure 1(b)), which are typically built around the same laser scanning platforms as other MPM systems. Traditionally, these systems have multiple detection channels, enabling simultaneous forward and backward collection. We have previously described the calibration process for SHG acquisition [32]. As THG is primarily forward emitted, as shown in Figure 1, we use a dichroic to separate out the SHG and THG signals. The most common excitation source used for SHG imaging is the Nd:YVO_4_ (532 nm pumped (titanium sapphire or ti:sapphire)) femtosecond oscillator, where these have tuning ranges of approximately 700-1000 nm. This spectral region is ideal for SHG imaging due to several considerations. Firstly, while scattering limits the penetration depth into tissue and decreases at longer wavelengths, the SHG conversion efficiency also decreases by about 3-fold over this range [33]. Moreover, this range enables simultaneous imaging of essentially all fluorophores via two-photon excitation [34]. We have previously shown that the SHG conversion efficiency increases at shorter wavelengths (at least measured to 780 nm), and while not exact, it followed the trend predicted by a two-state model [33]. However, collection of SHG at wavelengths less than 380 nm is limited by glass optics. THG on the other hand needs to be performed at wavelengths longer than about 1200 nm for optical transmission of the signal through glass optics. Laser sources for this purpose have included optical parametric oscillators, frequency-shifted fiber lasers, and Cr:forsterite [35, 36]. In practice, choosing the optimal wavelength for combined SHG and THG imaging experiments depends on available laser sources and emission filters that enable simultaneous excitation and detection.

Due to their ability to image structural features of tissues, polarization control is a critical aspect of both SHG and THG imaging. This is because these techniques are label-free and the contrast is subject to the constraints of the electric dipole interaction, where the best contrast is achieved when the molecular dipoles are parallel to the plane of laser polarization. This yields structural richness where specific information content and applicability will be discussed in latter sections. Here, we describe the general approaches to achieve precise polarization, either circular or linear, at the plane of focus in a SHG or THG microscope.

For SHG imaging, circular polarization is widely used as it simultaneously excites all orientations equally [32]. While circularly polarized light is readily attainable at the laser output with a quarter-wave plate, the resulting state at the plane of focus becomes elliptical due to non-45-degree reflections, birefringence, and strain in the dichroics and other optics in the path. We have shown how that can be corrected through the use of a half-wave plate before the quarter-wave plate that acts as a compensator [32]. Linear polarization can be similarly distorted and easily corrected through compensation by either a quarter- or half-wave plate or Babinet-Soleil compensator. We then use a liquid crystal modulator in the infinity space to either rotate the linear polarization or reverse the handedness of circular polarization for SHG-circular dichroism (SHG-CD) analysis [37]. Other polarization distortion correction approaches have been applied but are not as general as this combination of compensation and motion-free rotation in the infinity space [38]. Polarization-sensitive approaches to THG use similar instrumentation.

## 5. SHG Microscopy for Biomedical Applications

Over the last two decades, there has been an increasing interest in applying SHG and THG microscopies to image a wide range of tissues. In the next several sections, we will present emerging studies that have significantly contributed to the advancement of these tools for basic science and translational applications (Table 2).

**Table 2:** Summary of SHG-based techniques.

Method	Contrast source	Advantages	Disadvantages	Key information extracted
F/B ratio	Collagen fibrils	(i) Straightforward measurement with matched detectors	(i) Accuracy dependent upon calibration ratio (ii) Require tissues to be at least one scattering length thick	(i) Subresolution fibril organization/morphology (i.e., size and packing)
P-SHG	Collagen fibrils/fibers	(i) Motionless optics (ii) Pixel-based (iii) Differentiate between fibrillar collagen isoforms	(i) Difficult to translate to in vivo applications due to optical scattering (ii) Precise calibration of polarization needed (iii) Lengthy acquisition time	(i) Order of dipole moments and collagen helical pitch angle
SHG-CD	Alignment of chiral collagen within fibrils	(i) Quick measurement (ii) Sensitive (iii) Pixel-based	(i) Precise calibration of polarization needed	(i) Net chirality

### 5.1. Collagen Fiber Alignment

While the large majority of cancers are epithelial in nature, essentially all these tumors involve significant remodeling of the ECM both during early-stage disease and throughout progression [30]. These alterations can be in the form of increased collagen synthesis (desmoplasia), changes in morphology/alignment, and changes in collagen isoform expression, e.g., increased Col III or Col V synthesis. Historically, these ECM modifications have received little attention from pathologists, who mainly focus on cellular attributes via hematoxylin and eosin (H&E) staining (i.e., nucleus to cytoplasmic ratio) and expression of disease specific markers for diagnostics. Moreover, the eosin labeling is not highly sensitive to fibrillar collagen morphology. However, SHG microscopy, with its sensitivity/specificity to visualize collagen, is well suited for this task, and here we provide some seminal examples demonstrating this unique power.

**Figure 3:**
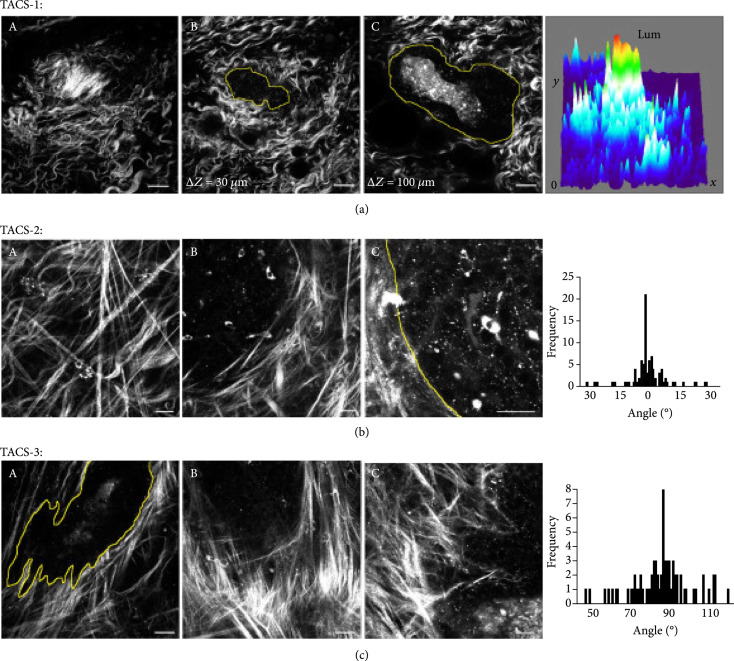
Characterization of tumor-associated collagen signatures (TACS) during disease progression in breast cancer models via SHG microscopy. Reproduced from Ref. [43] published under CC BY 2.0.

SHG microscopy has perhaps been particularly powerful in providing valuable insights into collagen remodeling in breast cancer [39-42]. It is well known that increased collagen density in breast tissue increases the risk of carcinoma; however, it is not directly causal, and conventional tools such as ultrasound cannot resolve the fibrillar structure [43]. To this end, in pioneering work, Keely and coworkers quantified collagen alignment to classify stages of remodeling during disease progression. Using murine tumor models, they identified three distinct and robust patterns they dubbed tumor-associated collagen signatures (TACS) [43, 44], defined by TACS-1, dense collagen accumulation around small tumors; TACS-2, elongated collagen fibers parallel to the tumor boundary; and TACS-3, collagen fibers normal to the tumor boundary, where the latter facilitate invasion (as demonstrated in Figure 3) and likely metastasis as well. Translating this study to human tissues in a tumor microarray, they found that TACS-3 was associated with disease recurrence and poor patient survival [40].

Similarly, Boppart and coworkers characterized the tumor microenvironment of untreated human breast tissues. Here, they custom built a portable nonlinear imaging system, integrating four modalities including SHG, THG, TPEF, and three-photon excited fluorescence [45]. This intraoperative platform enabled visualization of collagen alignment, tumor cell infiltration, blood vessels, mammary ducts, and lipids, with resolution and detail similar to those of the gold standard, H&E-stained histology. In invasive ductal carcinoma, they found that in response to tumor cell infiltration, the collagen was aligned parallel to the tumor cells. During both early- and late-stage disease, desmoplasia-associated collagen fibers were thick and straightened in comparison to thin and wavy in the tumor itself, and there was a distinct boundary between the cellular tumor and the desmoplastic regions. Additionally, during later stage disease, tumor cells infiltrated the desmoplasia-associated collagen region and shed extracellular vesicles, suggesting there was intracellular communication involved in the remodeling.

Pancreatic ductal adenocarcinoma (PDAC) has the poorest 5-year survival rate of all epithelial cancers. These tumors are characterized by a significant increase in stromal collagen density, making them well suited for investigation by SHG microscopy to explore whether analogous alignment changes to those in breast cancer occur in PDAC [46]. Indeed, Eliceiri and coworkers found that increased collagen alignment correlated with poor patient prognosis, where PDAC patients with low and high collagen alignment had median survival rates of 26.9 and 18.5 months, respectively [47]. Additionally, they found that this collagen reorganization was associated with an epithelial-mesenchymal transition expressed by PDAC cells and further that cancer-associated fibroblast markers were activated in the tumor microenvironment. Quantifying collagen reorganization and its relationship with such cellular processes provides critical information in terms of understanding disease pathology.

There have been other studies that examine the role of collagen alignment via SHG in several other epithelial cancers, including those of the ovary [48], prostate [49], and lung [50]. For example, SHG images of normal ovary and malignant tumors showed a vast morphological difference in the collagen assembly, where collagen fibers in the normal tissue were cross-hatched and randomly oriented in contrast to highly frequent wavy and aligned fibers in ovarian cancer [51]. Similarly, SHG images from human prostate cancer showed that collagen fibers in metastatic tumors were preferentially aligned in comparison to those that were still localized as well as to normal tissue [52]. Another group showed that *in vivo* non-small-cell lung cancer (NSCLC) xenograft models that overexpressed a cross-linking enzyme (LOXL1) resulted in higher collagen alignment, suggesting that increased cross-linking is a key component in NSCLC tumorigenicity [53].

In addition to solely assessing collagen alignment, cellular responses to these ECM modifications have also been investigated. For instance, Grzybowski et al. investigated trajectories of metastatic cancer cells and their noninvasive counterparts [54]. Here, they investigated metastatic potential using scaffolds of micropatterned lines, on which cells performed one-dimensional motions, mimicking the motility of cells migrating in 3D, and further are observed in metastasizing tumor cells *in vivo*. Using SHG and TPEF, they found that both metastatic and nonmetastatic cell lines infiltrated the dermis by following linear structural interfaces provided by parallel microtracks, blood vessels, or collagen bundles. However, the cellular kinetics were markedly different where metastatic cells were superdiffusive in zones away from the tumor while nonmetastatic cells reverted to ordinary diffusive motions.

### 5.2. SHG Polarization Analysis

**Figure 4:**
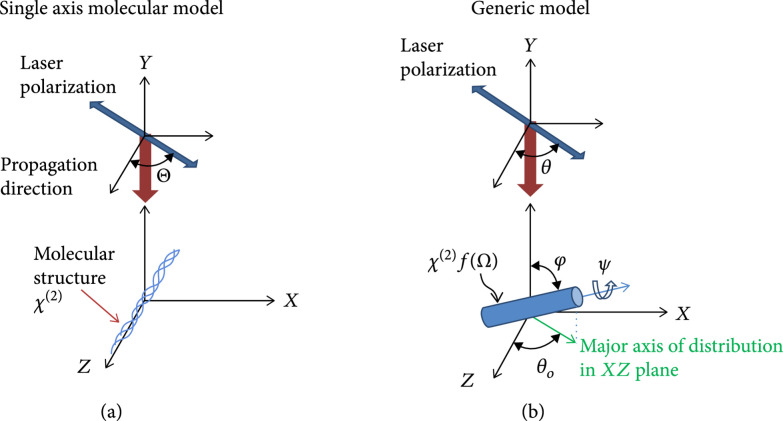
SHG polarization methods. Coordinate systems used in the (a) single-axis molecular and (b) generic models. Adapted from Ref. [57] with permission.

Imaging by SHG has additional richness beyond such visualization and analysis of the fibrillar morphology. The nonlinear susceptibility tensor, $\chi^{\left(2\right)}$, matrix elements contain information on the molecular and supramolecular structure that can be extracted utilizing polarization dependence on the excitation, SHG signal, or both. For example, we showed that measurements of the SHG intensity as a function of linear laser polarization can be analyzed to yield the *α*-helical pitch of well-aligned systems such as tendon and skeletal muscle, where this is dubbed the single-axis molecular model (Figure 4) [55-57]. In the general dipole expression, the SHG intensity can be defined as

(5)ISHGΘ=NP22=asin2Θ+bcos2Θ2+c2sin2Θcos2Θ,

where N represents the number density of the elemental dipoles and a, b, and c are numerical coefficients related to the matrix elements of $\chi^{\left(2\right)}$. If we assume both cylindrical and Kleinman’s symmetry, these coefficients can be reduced to: $a=N\chi_{ZXX}^{\left(2\right)}$, $b=\chi_{ZZZ}^{\left(2\right)}/\chi_{ZXX}^{\left(2\right)}$, and c=2, which are then related to the *α*-helical angle. Importantly, this analysis yields values in good agreement with structural biology studies. This model was improved upon by Dong and coworkers where they incorporated chiral and achiral components in their analysis and were able to differentiate Col I and Col II within the same tissue [56].

A limitation of this overall approach is the requirement of well-defined fiber alignment (e.g., found in tendon), where this condition is not met in most tissues. To address this problem, Brasselet et al. implemented a pixel-based generic model which analyzes the distribution of dipole moments within the focal volume [58]. We adapted this approach to reconstruct the linear polarization response described above and also determine the alignment of dipole moments within the focal volume (signal anisotropy), as demonstrated in Figure 4 [57]. For this purpose, 361 images were collected at each field of view, where 19 excitation and 19 emission angles were acquired every 10 degrees.

Further refinements and expansions of these ideas have focused on identifying specific collagen molecular attributes giving rise to the second-order nonlinear optical response. For example, using polarization in/polarization out (PIPO) and quantum calculations, Barzda et al. associated different parts of the collagen molecule with different tensor elements [59, 60]. This method allows for the extraction of the second-order susceptibility ratio, a measure of fibril distribution asymmetry, and fibril orientation (weighted average), both of which are important for the hierarchical organization of collagen. Furthermore, the triple helical tilt angle with respect to the fibril axis and overall fibril architecture within the 3D volume of each voxel can be deduced.

Additionally, the related SFG polarization-resolved microscopy can probe these aspects by using a combination of nonresonant NIR and resonant IR excitation. Using different approaches, SFG analysis has uniquely identified the chiral and achiral contributions from the collagen molecule. Specifically, using low-resolution microscopy, Knoesen et al. investigated the molecular origins of the second-order nonlinear response from collagen fibrils [61] and identified methylene groups associated with a Fermi resonance and carbonyl and peptide groups associated with the amide I band as the dominant contributors. Moreover, methylene groups are non-centrosymmetrically aligned and denoted by a tilt in the pyrrolidine rings of proline and hydroxyproline residues with respect to the fiber axis, resulting in a strong achiral contribution. In contrast, they found a strong chiral contribution from the helical disposition of carbonyl groups around the main collagen molecule axis in collagen I fibrils. Potma et al. investigated the source of the SFG contrast by tuning through known vibrational resonances [62]. Specifically, polarization analysis of the methylene groups revealed that the dominant SFG signal originates from the achiral $\chi_{yyy}^{\left(2\right)}$ and $\chi_{xxy}^{\left(2\right)}$ tensor elements, where these are preferentially enhanced over nonresonant components.

Lastly, SHG has been combined with circular dichroism (SHG-CD) implementation for a nonlinear analog of the standard spectroscopic tool used to study protein folding. This approach has sensitivity for studying chirality of protein assemblies; however, SHG-CD is a coherent process and does not require absorption like conventional CD. In brief, images are collected using left- and right-handed (LH and RH) circularly polarized (CP) laser excitation and intensities of the two images are measured and denoted by

(6)ISHG-CD=ILHCP−IRHCPILHCP+IRHCP/2,

where $I_{\text{RHCP}}$ and $I_{\text{LHCP}}$ refer to SHG pixel intensities from RHCP and LHCP images, respectively. This normalized difference can be related to collagen attributes such as the triple helix chirality and the out-of-plane tilt angle [63].

Collectively, these polarization schemes, sometimes generally denoted P-SHG, have received considerable attention over the last several years [59, 64, 65]. Here, we highlight a subset of applications using P-SHG.

#### 5.2.1. Cancer Applications

We have used the generic pixel-based model [58] described above to probe the supramolecular structure of collagen in ovarian cancer [57]. This study was undertaken as previous studies based on immunostaining had suggested that the Col III isoform was upregulated in high-grade disease relative to normal stroma [20]. As the respective *α*-pitch angles of the Col I and III isoforms are different by about 2 degrees, in principle, these can be delineated by the single-axis molecular model. We validated this approach using a series of self-assembled collagen gels, ranging from 0 to 40% Col III, with the balance comprised of Col I and delineated structural differences via P-SHG, where the latter had a higher pitch angle, consistent with structural biology analysis [57]. However, the pitch angle in human tumors was lower than normal and inconsistent with a Col III increase [65]. We found lower anisotropy (i.e., dipole alignment within fibrils) and lower SHG-CD in high-grade disease relative to normal tissues, where this is consistent with either rapidly degraded or incorrectly synthesized collagen.

Barzda and researchers used PIPO to quantify structural changes of collagen in breast and lung cancers [50, 66]. Examining three pathologic types of invasive breast cancers, they found that tumors overexpressing estrogen, progesterone, and human epidermal growth factor receptors all resulted in significant changes within the collagen triple helix and/or fibril organization [66]. In their most recent study investigating different stages of non-small-cell lung carcinoma, they showed that during tumor progression, the submicron collagen architecture is more disorganized and fragmented [50]. Additionally, the remodeled collagen fibers were straighter and more aligned.

#### 5.2.2. Fibrosis

**Figure 5:**
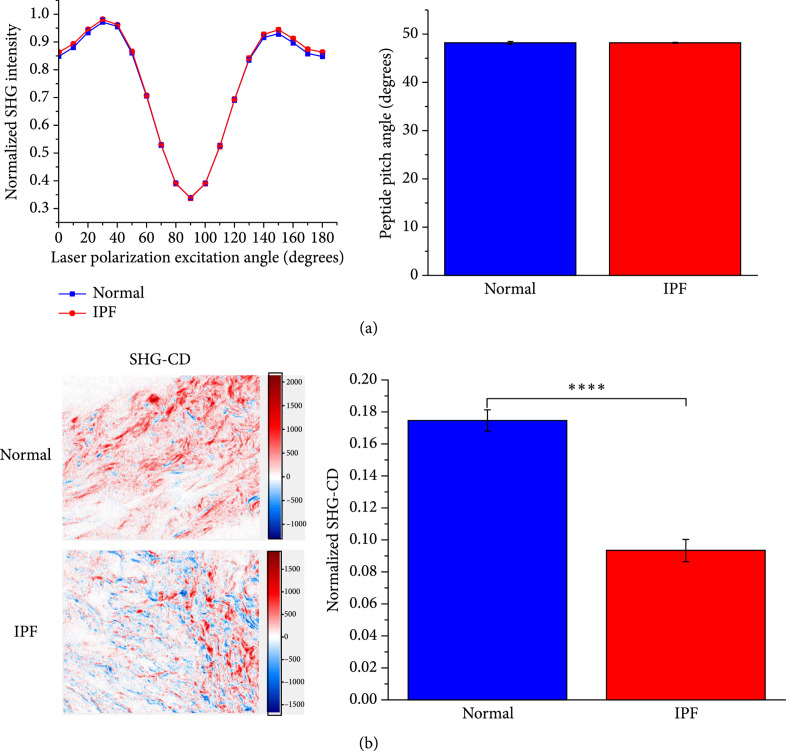
Polarization-resolved studies on human lung tissues. (a) SHG intensity and peptide pitch angle and (b) SHG-CD images and normalized SHG-CD for normal and IPF. Adapted from Ref. [69] published under CC BY 4.0.

Similar to ovarian cancer, it has been suggested by immunostaining that Col III is upregulated in idiopathic pulmonary fibrosis (IPF) [67, 68]. Likewise, our pixel-based SHG analyses also revealed no observable differences in the *α*-helical pitch angle between normal and IPF human tissues [69]. However, SHG-CD analysis showed that the chirality of the triple helix was significantly decreased (almost twofold) in the diseased state. Further response, the SHG-CD also showed a more random distribution of fiber polarity, where taken together these observations are consistent with incorrectly assembled collagen in IPF (Figure 5). The question as to why P-SHG did not show differences in the *α*-helical pitch angle in both IPF and ovarian cancer remains unanswered. However, we note the literature suggestions were based solely on immunostaining, where the available antibodies are not specific to Col III.

Similarly, Huang and researchers utilized P-SHG to analyze changes in collagen isoform balance and fibril orientation during progressive fibrosis in bile-duct-ligation (BDL) rat liver models [70]. BDL samples harvested between 0 and 6 weeks showed an increase in collagen deposition with more randomly oriented fibers. Furthermore, these studies revealed that the Col III/Col I ratio significantly increases during disease progression, suggesting that collagen type III is an important contributor in the severity of liver fibrosis.

#### 5.2.3. Cornea

Schanne-Klein’s group demonstrated the novel capability of P-SHG and advanced data processing to visualize and accurately orient submicron spatial features of the structural organization in human corneal stroma via backward-detected SHG [71]. While not precisely providing structural information about corneal stroma, the resulting backward-collected P-SHG intensity was most significant when collagen fibrils were aligned with the incident excitation field. As a first step toward *in vivo* imaging, they imaged *ex vivo* whole porcine eyeballs and retrieved information from different lamellar domains through the entire 800 *μ*m cornea thickness. Although forward directed signals could not be detected with this configuration, their results showed that lamellar domains were smaller in the anterior vs. posterior stroma, consistent with physiological data. Additionally, they successfully performed *in vivo* imaging of rat cornea using epi-detected P-SHG and were able to determine orientation and $\rho =\beta_{XXX}/\beta_{XYY}$, which is reflective of the anisotropy of nonlinear responses from lamellar domains of collagen fibrils, throughout the entire thickness (~150 *μ*m). These studies on cornea present evidence revealing the power of P-SHG to probe the submicron stromal collagen features despite dynamic movements and its potential to be a useful diagnostic tool for diseases that undergo collagen remodeling.

In another study in ocular samples, Bueno et al. used Stokes-vector based polarimetry and SHG-CD to assess internal organization and explore the relationship between diattenuation, which contains global information on polarization sensitivity, and external collagen organization [72]. Here, they collected four polarimetric SHG images corresponding to left circular and linear horizontal and two elliptical incident polarization states from 14 different ocular samples. Their results revealed that increased external fiber distribution resulted in a higher diattenuation of the polarization response sensitivity. Furthermore, this suggested that this behavior is general and does not depend on the angle between the fiber axis and incident polarization. Moreover, in partially and quasi-aligned samples, they found a strong structural correlation between collagen fiber orientation and direction of maximum intensity, suggesting that selecting the appropriate incident polarization could increase SHG signal obtained from samples that would otherwise present with low contrast. Additionally, SHG-CD values and hyperpolarizability ratios ($\rho =\beta_{XXX}/\beta_{XYY}$) had a linearly dependent relationship, indicating that collagen chirality is highly associated with internal (i.e., molecular) collagen fibril organization.

#### 5.2.4. P-SHG and Optical Clearing

While powerful in probing structural changes in tissues, P-SHG techniques have a fundamental limitation in imaging depth. For example, we showed that these responses become largely scrambled within 1-2 scattering lengths (~20-50 microns) in both tendon and skeletal muscle [73]. It was previously reported by Wang using Mueller matrix analysis that birefringence (found in collagenous tissues) results in asymmetric scattering and can cause a rapid depth-dependent loss of polarization [74]. Similarly, Schanne-Klein et al. performed an in-depth analysis specific to SHG considering effects including scattering, birefringence, and diattenuation and verified that birefringence played a major role in depolarization [75].

To help overcome the inherent depth limitations in turbid tissues while maintaining high resolution coupled with polarization analysis, SHG imaging can be combined with optical clearing [76]. Here, samples are placed in a high refractive index, hyperosmotic reagent (e.g., glycerol, sugars, or sugar alcohols) to increase its transparency. We have shown that this reduces the scattering coefficient by ~5- to 20-fold and P-SHG of tendon and muscle fully retained the correct polarization signatures [77]. Indeed, most of the studies reviewed above were either performed on thin sections (less than one scattering length) or were optically cleared with 50% glycerol.

#### 5.2.5. THG Polarization Microscopy

Until recently, polarization-resolved THG (P-THG) was infrequently used for biological imaging. Many of these imaging experiments use linearly polarized laser light because the induced third harmonic polarization vanishes in isotropic media excited with circularly polarized light, even if heterogeneity exists [78]. However, strongly birefringent media can produce efficient THG with both circularly and linearly polarized light and provide new structural information. For example, using linearly and circularly polarized THG (P-THG), Bautista et al. were able to identify and discriminate between lipid droplets (LDs) with different lipid compositions. First, they imaged synthetic enriched LDs followed by a macrophage model and P-THG successfully discriminated between the different lipid compositions due to their intrinsic ordering [79]. Similarly, Beaurepaire et al. used P-THG to image multilamellar vesicles, validating its use to probe lipid orders [80]. Applying this technique to a more complex tissue environment, they imaged human skin biopsies and found that THG signal was localized near interfaces with dried, dead corneocytes which consisted of stacked lipid bilayers.

More detailed analysis of the polarization response can provide additional richness. Barzda et al. implemented P-THG by combining nonlinear Stokes-Mueller polarimetry with THG microscopy [81]. Investigating two common symmetry classes, an isotropic and cylindrical case, they successfully recovered all eight accessible $\chi^{\left(3\right)}$ tensor components directly from measured data, where these values were comparable to those obtained from different models. In a more recent study, Beaurepaire et al. developed a fast P-THG microscopy technique to quickly modulate excitation polarization combined with Fourier-based analysis [82]. Using this approach, they were able to probe molecular ordering in deforming multilamellar lipid structures, examine biomineralization of flowing particles in zebrafish, and also detect birefringence *in vivo*.

### 5.3. SHG and THG Physics-Based Approaches

Both SHG and THG are coherent processes, where there is a spatial and temporal relationship between the excitation and generated signal based on phase-matching considerations. This results in a distribution of forward and backward emitted components that depend on the tissue structure. While this presents additional experimental challenges relative to fluorescence, which is incoherent and equally emitted over 4*π* steradians, as we will describe below, there is a wealth of structural information in the spatial emission pattern in these harmonic modalities.

Expanding upon the framework of Mertz and Moreaux [83], we developed an empirical model to predict trends of the forward and backward emitted components and relative SHG intensities as a function of collagen fibril size and packing on the axial size scale with respect to $\lambda_{\text{SHG}}$ [84]. This emission pattern arises from the phase mismatch (Δk), which is defined by $\Delta k=k_{2\omega }-2k_{\omega }$, where $k_{2\omega }$ and $k_{\omega }$ are the wave vectors for the SHG and incident photons, respectively. For ideal phase matching, Δk=0, which can be seen in uniaxial crystals and interfaces, SHG emission is 100% forward directed and copropagates with the laser with infinite coherence length. However, such conditions do not exist in biological tissues. This results in a distribution of forward and backward components due to the need to conserve momentum, where the SHG intensity is modulated by a sinc^2^ function of Δk. A larger phase mismatch results in both a lower forward-backward ratio, which we denote the creation ratio or emission directionality, $F_{\text{SHG}}/B_{\text{SHG}}$, and weaker SHG intensity. In this treatment, “domains” or large or packed fibril structures axially aligned on the order of $\lambda_{\text{SHG}}$ result in predominantly forward SHG, whereas smaller and/or more random structures with larger Δk values are associated with backward directed SHG (although $F_{\text{SHG}}/B_{\text{SHG}}\geq 1$) [27].

While this emission directionality contains potentially valuable subresolution structural information, in general, it is not directly measurable in a tissue imaging experiment, as the forward and backward components become convolved with scattering. The SHG emission and scattering cannot be decoupled analytically in the quasi-ballistic regime (~few scattering lengths) and we must use Monte Carlo (MC) simulations based on the bulk optical properties (scattering coefficient ($\mu_{\text{s}}$), scattering anisotropy (g), and absorption coefficient ($\mu_{\text{a}}$)) to isolate $F_{\text{SHG}}/B_{\text{SHG}}$. This scheme can also be used to extract the relative SHG conversion efficiencies in tissues, which are related to the collagen density as well as the phase mismatch.

We have used this approach in the analysis of several tissues, including connective (tendon, muscle, cartilage, and skin), ovarian, and lung tissues [51, 69, 85-87]. Our emphasis has been on delineating structural differences in normal and diseased states, and here, we will highlight our efforts on human ovarian cancer. In initial studies, we found that high-grade serous ovarian cancer (HGSOC) had a lower $F_{\text{SHG}}/B_{\text{SHG}}$ than normal stroma, which was in agreement with predictions of our phase-matching model based on the respective TEM data [51]. We further applied this analysis to visualize heterogeneity within the tissues, by examining the emission directionality in small “pixel patches” on the size scale of ~10×10 microns [63]. Interestingly, we found very similar $F_{\text{SHG}}/B_{\text{SHG}}$ values across the image for HGSOC, whereas these were widely distributed for low-grade serous ovarian cancer. We speculated that this could arise from genetic differences between the diseases. For example, HGSOC is largely characterized by p53 alterations where in contrast low-grade disease is comprised of several other mutations [88, 89].

Performing this analysis over a range of wavelengths provides additional richness, as this affords comparing the SHG responses to the distribution of fibril sizes. For example, a weak wavelength dependence of $F_{\text{SHG}}/B_{\text{SHG}}$ and conversion efficiency would indicate that the fibrils are much smaller than the excitation wavelength. We used this approach to compare HGSOC, low-grade disease, and benign tumors over the spectral range of 780-1200 nm [90]. We found a fairly flat response for HGSOC, where the other tumors were characterized by higher $F_{\text{SHG}}/B_{\text{SHG}}$ values at longer wavelengths. This indicates that these tissues are characterized by larger harmonophore assemblies where the phase mismatch is reduced at longer wavelengths.

We also determined the wavelength-dependent relative conversion efficiencies for these tissues. The efficiency is a compounded effect of $\chi^{\left(2\right)}$ and the phase mismatch, which can have different spectral dependencies. Based on the two-state model [91], the susceptibility will decrease at longer wavelengths, where in contrast the phase mismatch will depend on tissue structure. Here, we found that the HGSOC conversion efficiency decreased at longer excitations and in fact behaved like we reported previously for tendon [33]. Interestingly, both tissues are characterized by regularly sized, uniformly packed fibrils that are ~60 nm in diameter. In comparison, the other tumors displayed higher conversion efficiencies at longer wavelengths due to larger fibrils providing improved phase matching (and higher $F_{\text{SHG}}/B_{\text{SHG}}$). In sum, these analyses based on coherence provide subresolution assessments, especially in terms of identifying structural differences between different tissue states.

**Figure 6:**
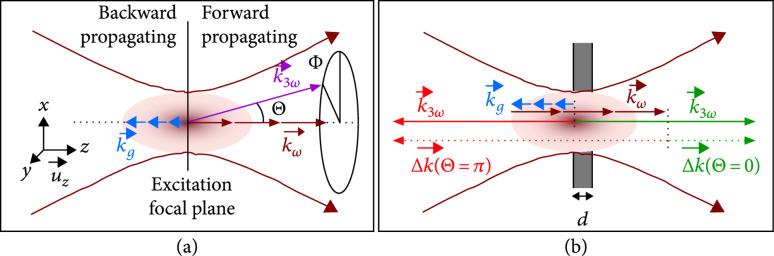
THG emission efficiency. (a) Directional emission with respect to the excitation plane. (b) Forward and backward wave vector mismatch on a slab-like sample. Reproduced with permission from Ref. [92] © The Optical Society.

While closely related to SHG, the THG emission directionality patterns in biological media have received considerably less attention. In early work, Mertz and Moreaux showed that for the case of ideal phase matching, the emission was in the form of a solid cone [83]. As depicted in Figure 6, the THG phase-matching conditions are denoted by $\Delta k=3\left(k_{\omega }+k_{\text{g}}\right)-k_{3\omega }$, where $k_{\text{g}}$ describes the Gouy phase shift experienced inside the sample and $k_{3\omega }$ and $k_{\omega }$ represent the wave vectors for the THG and incident photons, respectively. Beaurepaire et al. showed that most biological objects do not typically produce significant backward directed THG because the backward directed phase mismatch is typically two orders of magnitude larger than that of the forward [92]. Thus, for efficient backward THG emission, the Fourier spectrum needs to be comprised of large Δk values that correspond to structures smaller than 100 nm in the z direction, or the sample layer thickness must equate to backward radiating dipoles constructively interacting. Translating these theories to biological tissue imaging, THG was undetectable in the epi-direction in thin rat lung tissues in comparison to thick ones, illustrating that most of the collected signals arose from scattering of forward emitted components rather than direct backward coherent emission.

### 5.4. Image Analysis and Machine Learning

Over the past decade, image processing and machine learning tools have significantly advanced and have been shown to not only delineate but extract morphological features from SHG images from different types of tissues. While these tools have not yet been widely adopted or used clinically, we will discuss how they have enhanced our understanding of tissues especially in different diseases and how they may eventually be used for this purpose.

Several classes of image processing techniques have recently been utilized to quantify and/or classify SHG and THG images based on the tissue morphology. While segmentation methods based on thresholding are the simplest to employ and have been used to score fibrosis in the kidney [93] and liver [94], these techniques are only sensitive to intensity and not tissue architecture. To improve upon this, the use of signal processing tools including fast Fourier transforms (FFT) and grey level co-occurrence matrix (GLCM) has been explored. These techniques have associated outputs including alignment and correlation coefficients (e.g., energy and entropy). For example, we combined FFT, GLCM, and CT-FIRE to classify collagen architecture in normal fallopian tubes, high-grade ovarian and serous tubal intraepithelial carcinomas [95]. While each approach revealed only subtle differences between the two groups, combining the respective metrics in a linear discriminate analysis resulted in higher classification accuracy.

Image analysis tools have been developed to determine changes automatically and robustly in collagen morphology seen in SHG images. Global image tools like 2D Fourier transforms do not provide region-specific information such as changes in alignment around tumor boundaries seen in breast cancer and PDAC. However, local transforms have greater potential in this regard. For example, Eliceiri et al. implemented the fast discrete curvelet transform (CT) to identify such regions of local alignment by determining the angle of collagen fibers with respect to a user-defined or computationally segmented boundary [96]. This software is freely available as the CurveAlign ImageJ plugin. They also developed the CT-FIRE program that utilizes both the curvelet transform and a fiber extraction (FIRE) algorithm [97] to extract descriptive collagen fiber metrics (i.e., width, length, straightness, and angle) on an individual fiber basis [98].

Analyzing collagen fiber orientation is another area of interest in tissue characterization, especially in terms of characterizing the 3D ECM architecture. A powerful tool for this purpose was developed by Georgakoudi et al. that rapidly and accurately identified intensity variations in all directions within voxels of adjustable volumes [99, 100]. To demonstrate its applicability, they characterized collagen fiber organization in simple models and then more complex articular cartilage and breast cancer tissues. Their results successfully differentiated the layers of collagen organization in the different regions of cartilage and showed a decrease in 3D directional variance in breast cancer tissues, where lower directional variance corresponds to highly aligned fibers. Examining pancreatic tissues, they uncovered the potential for a decrease in 3D directional variance to reveal peritoneal metastases.

While these local and transform approaches have been successful in analyzing tissue structure, they do not specifically utilize machine learning (ML) techniques. We employed a ML texture analysis method, known as textons, to distinguish different classes of ovarian cancers [101]. Using unsupervised learning via k-means clustering, textons (or repeating features) are identified by convolving small pixel patches of images with a 3D filter set of different orientations and scales. In the supervised learning classification stage, differences between the texton distributions of test and training data are evaluated by Gaussian weighted nearest neighbor comparison, which then are used to generate ROC curves to calculate classification accuracy. Implementing this tool to evaluate SHG images of normal ovary, high-risk ovarian stroma, benign and endometrioid tumors, and low- and high-grade serous ovarian cancers, we successfully classified the different classes with ~83-91% accuracy. Importantly, the same analysis on 2D data sets resulted in significantly lower accuracies (~70%), stressing the importance of examining the 3D ECM architecture.

Given the rapid development of deep learning techniques, these are beginning to be applied to harmonic microscopy images. Unlike machine learning, which requires labeled input data to train algorithms, deep learning relies on multilevel hierarchical layers in neural networks to train itself with little to no supervision or intervention. Additionally, deep learning allows for transfer learning, in which neural networks are trained on one sort of data set and can be applied to a different data set. Using this idea, Yu and researchers applied an automated deep learning-based algorithm to score images of liver fibrosis [102]. Based on the pathological scoring of the different fibrotic stages, they generated classification models using different supervised techniques, namely, convolutional neural networks (CNN), artificial neural networks, multinomial logistic regression, support vector machines, and random forests. By extraction of morphological and textural features and classification, they showed that the pretrained CNN produced accuracies between 85 and 95%, comparable to those from the other techniques. This suggests that transfer learning approaches may be used for weakly correlated and even unrelated images to overcome the current deep learning limitation of needing large image libraries. This is important as most often microscopy, especially on human tissues, is performed in the “data poor” regime.

We have provided a brief overview of the different approaches being used for image analysis and machine learning classification of SHG data. Table 3 summarizes the advantages and disadvantages of these methods. Tools such as GLCM and FFT are readily applicable to even small data sets for comparing image features. Typically, larger data sets are needed for classification schemes such as textons and the CNN-based deep learning methods. However, these in general are more powerful as they do not rely on simple morphological features such as fiber size and alignment, but rather on the overall features in the images. We note that new libraries need to be generated for each new tissue type/class to be analyzed.

**Table 3:** Comparison of image analysis/machine learning techniques. Adapted from Ref. [103].

Analysis tool	Pros	Cons
2D FFT	(i) Boundaries not required (ii) Integrated in a variety of image analysis tools (i.e., FIJI and Matlab)	(i) Global approach (ii) Difficult to detect small alterations in tissues with random fiber alignment
Curvelets	(i) Extracts collagen fiber in relation to defined boundaries; extremely useful in breast cancer studies	(i) Needs tumor/cell boundary for accuracy; may not be readily translated to other diseases
GLCM	(i) Simple intensity-based approach (ii) Widely available in multiple image analysis platforms (i.e., FIJI and Matlab)	(i) Highly variable and dependent on collagen coverage (ii) Low sensitivity and specificity in most applications to date
Textons	(i) Filters independent of fiber and image characteristics	(i) Requires large image library (ii) Does not identify discrete features (iii) Computationally intensive: making it difficult to integrate
3D-voxel	(i) Translatable to multiple imaging modalities (ii) Rapid and highly accurate (iii) Voxel-wise information	(i) Requires fiber-like features (ii) May not be well suited for thick tissues
Deep learning	(i) Less tedious and more efficient (ii) Independent of specified image characteristics (iii) Implementation of transfer learning enables use of small data sets	(i) Can require large image library (ii) Complex training of neural networks

### 5.5. Other Biological Applications

SHG microscopy has uncovered a profound remodeling of collagen in the ECM in many cancers and diseases, but in order to truly understand disease biology, it is also important to explore how these collagen alterations impact cellular interactions. For this reason, several labs have used SHG beyond its visualization capabilities and have revealed some downstream effects of collagen alterations including tumor growth, therapeutic response, and new matrix synthesis.

#### 5.5.1. Imaging Chemotherapy Activity

While SHG imaging has been widely used to study ECM alterations in cancer, only recently has the chemotherapeutic response of tumor-associated collagen been examined. Shirmanova et al. used SHG and metabolic imaging to monitor tumor response to chemotherapy (i.e., cisplatin, paclitaxel, or irinotecan) *in vivo*, in mice implanted with colon cancer cells [104]. In untreated control tumors, the collagen to cellular ratio significantly increased. Interestingly, different chemotherapeutic agents resulted in the specific tumor-associated collagen (TAC) multidirectional alignment. For example, cisplatin and paclitaxel resulted in an early increase in SHG signal (increased collagen content or desmoplasia) whereas this was not impacted by irinotecan treatment. Collectively, their results suggest that alterations in cellular metabolism and collagen architecture occur prior to cell morphological changes.

These results are similar to those found in the studies of Wu et al. where they used autofluorescence and SHG to study chemotherapeutic response in advanced human breast cancer [105]. Here, they assessed the optical redox ratio, SHG to fluorescence ratio, and collagen density and orientation and found changes in collagen shape and alignment. Specifically, in comparison to normal and postchemotherapy, the collagen fibers were much straighter in the remaining carcinoma tissues. Additionally, while collagen degenerates during tumor progression, it regenerates after chemotherapeutic application, resulting in a fibrotic response.

#### 5.5.2. Imaging Tissue Engineering Scaffolds

Conventional techniques such as immunofluorescence are not often successful in capturing the dynamic ECM environment of tissue engineering (TE) constructs. However, SHG is well suited for examining collagen synthesis from live cells in scaffolds, especially in conjunction with fluorescent markers. Here, we provide a couple examples of these possibilities.

Given that congenital heart defects (CHD) remain the leading cause of death in infants and children, TE approaches have recently been explored since surgical procedures have been widely ineffective at recapitulating the native heart anatomy and function. However, these TE applications have not examined fetal ECM organization and how it effects cardiac regeneration. To this end, Georgakoudi and coworkers explored the impact that such tissue has on the expansion of cardiomyocytes *in vitro* [106]. Here, they engineered ECM-coated substrates and seeded them with fetal, neonatal, and adult cardiomyocytes. They assessed the abundant proteins in each treatment group and found that fibronectin was the most prevalent in fetal and neonatal hearts whereas collagen I was the most dominant in adults. SHG images of decellularized hearts revealed that fetal ECM was associated with small collagen fibers resulting in significantly lower SHG intensity, which may explain why fetal hearts required less potent treatment and much shorter digestion times than adult tissues (1 h vs. up to 48 h). Additionally, cardiomyocytes not only adhered and expanded better on fetal ECM but they were also positive for mitosis, suggesting that proliferation may be better promoted as well.

Enejder and coworkers simultaneously captured SHG and coherent anti-Stokes Raman scattering (CARS) images to visualize microporous bacterial cellulose scaffolds seeded with osteoprogenitor cells to investigate the correlation between cells and new collagen synthesis [107]. First, they compared SHG and histology images of scaffolds one and seven days after seeding, with both methods similarly showing a significant increase in collagen content in the longer term scaffolds. Further, their results showed that scaffold material was capable of initiating osteogenesis due to the rather immediate (first days of growth) production of collagen by osteoprogenitor cells. Additionally, they found collagen fiber networks inside compact dense cellular regions within cellulosed micropores, which are important for new bone formation.

Cellular dynamics (i.e., orientation, adhesion, and migration) are essential to fully attain physiologic function, making their regulation extremely important for TE and cell therapy studies [108]. Researchers have employed physical stimuli and have shown that, in response, cells cultured in 2D and 3D environments change shape and orientation. An alternative mode is electrical stimulus, which is simple, flexible, feasible *in vivo* and *ex vivo*, and similar to physiological electrical activities in normal biological processes like embryonic development and wound healing [108]. Although cell behavior in response to electrical stimulus is well characterized in 2D cultured cells, this effect in a 3D matrix remains largely unknown. To this end, Cho et al. coupled SHG imaging and autofluorescence to evaluate the impact of electric stimulation on collagen and cell orientation and alignment in 3D collagen scaffolds [108]. While they found that electrical stimulation in collagen gels embedded with fibroblasts resulted in preferential collagen and cell alignment normal to the direction of the applied force, rat mesenchymal stem cells (MSCs) only showed minimal changes in cell orientation. Moreover, prior to stimulation, collagen fiber bundles were denser and more tightly packed with embedded MSCs, suggesting that the tight network prevented changes in orientation. This study reveals the power of SHG imaging to monitor specific cell-induced dynamic interactions in a 3D environment, which is imperative for optimizing tissue engineering approaches.

#### 5.5.3. Imaging Col II in Cartilage

While this review has been highly focused on Col I, the Col II isoform is the primary matrix component of cartilage, including that of the patella. It is also fibrillar and, while morphologically distinct from Col I in structure, produces easily imaged SHG contrast. Historically, the cartilage in the patella is grouped into three zones delineated primarily by H&E staining. By examining the physical/structural depth-dependent ECM modifications in articular cartilage, we identified unique SHG signatures at both the fiber and fibril assembly in these three histological zones [85]. Interestingly, through SHG forward/backward analysis, we found that the middle zone contained the most organized fibril assembly, which is in strong contrast to the histological analysis of transverse sections which showed a disorganized structure.

**Figure 7:**
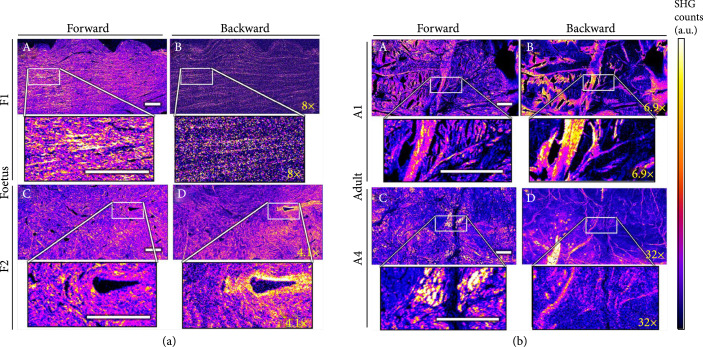
Forward and backward SHG images. Images of (a) fetal and (b) adult equine meniscus. Adapted from Ref. [109] published under CC BY 4.0.

In another study, Légaré et al. used SHG forward/backward analysis and P-SHG to examine the collagen architecture in equine fetal and adult meniscus [109]. As shown in Figure 7, they found large differences in not only the collagen architecture but also fibril orientation as a result of aging. In comparison to adults, the fetal menisci displayed a lower F/B ratio, consistent with a less organized fibrillar structure. Additionally, through P-SHG, they showed that adult menisci had thick homogeneous clusters of fibrils oriented in the same directions.

## 6. THG Microscopy Biological Applications

THG microscopy is well suited for investigating complex system biology and when combined with other nonlinear modalities like SHG and TPEF allows for the precise mapping of several biological components. Here, we highlight some important applications of this imaging modality.

### 6.1. Developmental Biology

**Figure 8:**
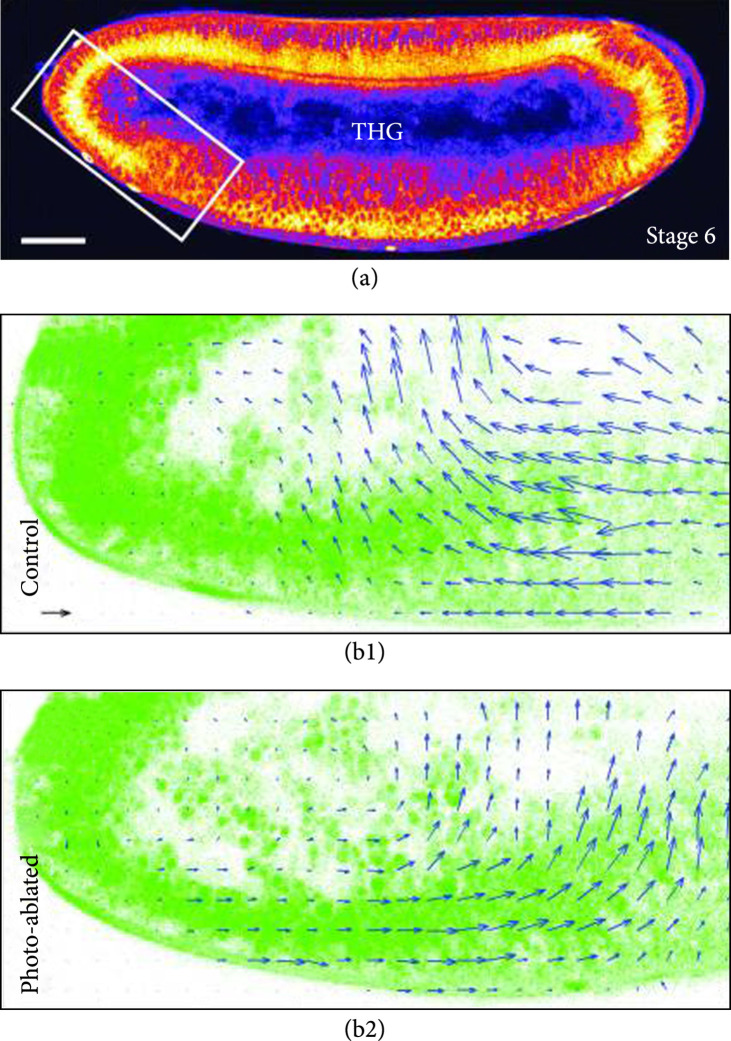
Photoablation of Drosophila embryos. (a) THG equator image of unstained WT embryo. Velocimetric analysis of morphogenic movements of the (b1) control and (b2) photoablated embryo. Reproduced from Ref. [110] with permission.

Perhaps the largest application of THG has been imaging aspects of developmental biology. This is because cell membranes and other organized structures with a change in refractive index are abundant in developing embryos. For example, Beaurepaire et al. were able to modulate, visualize, and quantify the complex biomechanical movements in Drosophila embryos *in vivo* by combining controlled intravital laser ablation and multimodal nonlinear microscopy (TPEF and THG) [110]. Here, they found that multiphoton ablation created a local microdissection within developing embryos, without significantly disrupting the surrounding cytoskeleton behavior, and THG microscopy successfully captured the resulting structural architecture (Figure 8). In addition, velocimetric data revealed information on the tissue dynamics, yolk internal structures, and their overall continuity. These experiments can be performed with strong biological viability due to the use of longer wavelengths (>1200 nm).

In a later study, they examined real-time micrometer-sized lipid bodies *in situ* via THG, allowing them to quantify lipid metabolism, image and track single lipid bodies in embryos, and map their distribution in intact animal and plant tissues [111]. Imaging hepatocytes isolated from rat liver under normal and regenerating (24 h after partial hepatectomy) conditions, they found that THG successfully detected differences in size and aggregation of lipid bodies which was significantly different between the two treatment groups. In addition, THG, SHG, and TPEF images of murine lung tissue allowed for visualization of the interactions between lipid bodies, the ECM, and cellular redox state, which may provide insight into physiological processes responsible for organ development and enable further assessment of altered fat metabolism observed in diseases such as diabetes and atherosclerosis.

### 6.2. Skin Cancer and Aging

**Figure 9:**
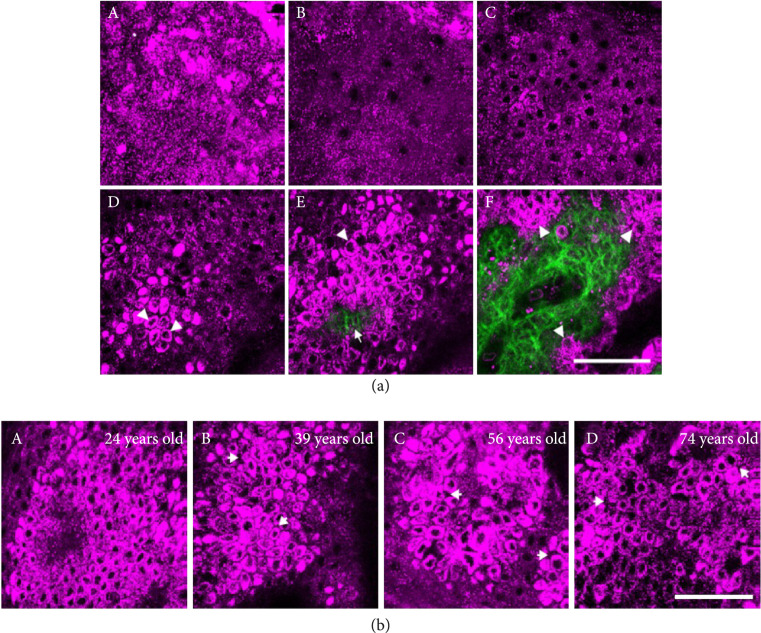
In vivo images of the skin. (a) SHG and THG images from different depths (regions) of the volar forearm of a 24-year-old subject. (b) THG images of epidermal basal cells from (A-D) 24-, 39-, 56-, and 74-year-old subjects, respectively. Adapted with permission from Ref. [112] © The Optical Society.

Combining THG and SHG microscopies, Sun and coworkers have performed numerous studies imaging skin *in vivo*. One area of interest has been imaging changes in morphology due to aging, where THG and SHG visualize epidermal cellular morphology and dermal collagen, respectively [112]. As shown in Figure 9(a), THG contrast specifically captured differentiated changes in nuclear size, cell size, and cell density at different depths including the stratum corneum, stratum granulosum, stratum spinosum, and stratum basale. They found that basal cells were oval-shaped and organized in a honeycomb fashion in young skin and over time they become irregularly shaped ultimately losing their overall organization (Figure 9(b)). In addition, basal keratinocyte morphology was found to be a good scoring criterion for skin aging as both cellular and nuclear sizes were larger in elderly subjects in comparison to younger ones.

They further demonstrated the combined applicability of THG and SHG for analysis of noninvasive virtual skin cancer biopsies [113]. Here, they were able to identify cellular cytoplasm of keratinocytes, melanocytes, red blood cells, and fibroblasts via THG and collagen and elastin fibers by SHG and autofluorescence, respectively. This multimodal approach revealed an aggregation of melanoma cells in the epidermis in melanoma and also nodules of tumor cells enclosed by collagen fibers in pigmented basal cell carcinoma (BCC). More recently, to better understand THG sources in the skin, they decoupled the overpowering effects of melanin from other components using vitiliginous skin [114]. Furthermore, they assessed therapeutic efficacy of ultraviolet B (UVB) therapy in treating vitiligo to restore pigmentation and saw an increase in THG intensity for 7 out of 15 patients after 24 weeks of UVB treatment. In fact, in some cases, the intensity was comparable to that from normal basal keratinocytes.

### 6.3. Brain Imaging

**Figure 10:**
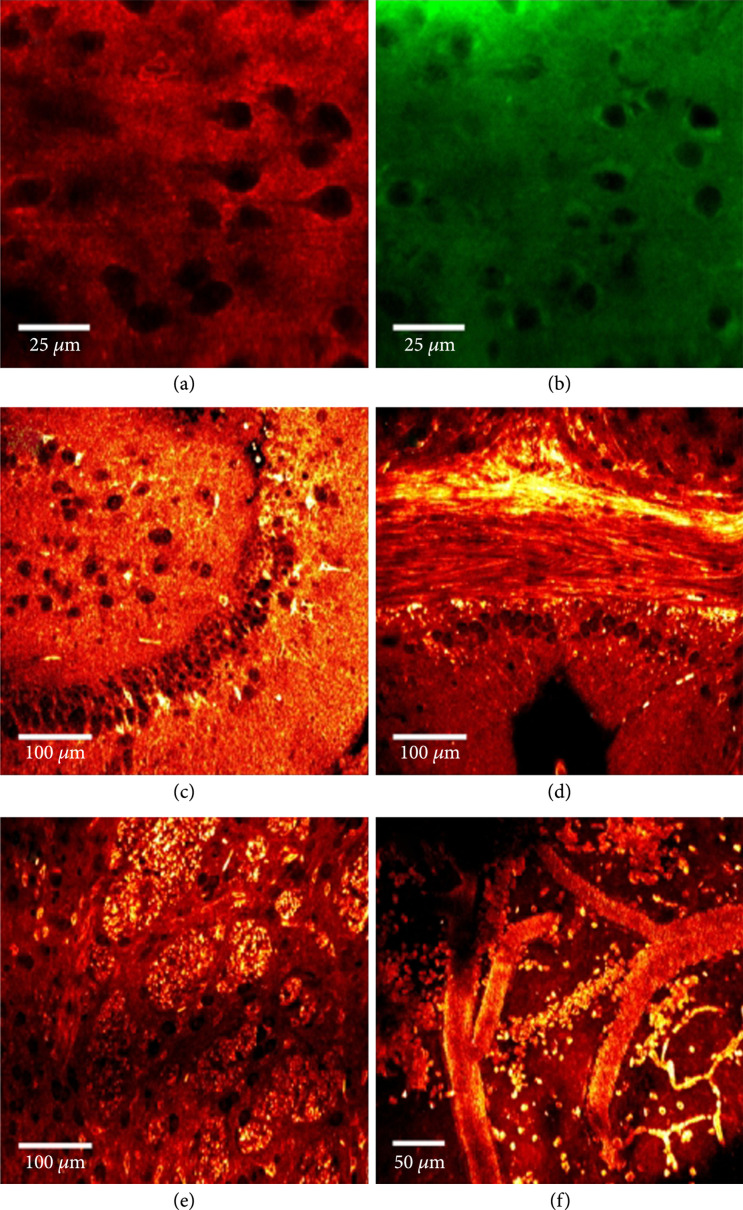
THG images of living brain tissue. (a) THG image of mouse neocortex. (b) TPEF image of Nile Red-stained lipid from same location as (a). (c) THG image of rat dentate gyrus. (d) THG image of mouse corpus callosum. (e) THG image of striatum in the mouse brain. (f) THG image of blood vessels from the cerebral cortex of a live, anesthetized mouse. Reproduced from Ref. [115] with permission.

THG is also capable of imaging lipid structures in the brain; for example, Groot and coworkers simultaneously imaged neurons, white-matter structures, and blood vessels, all of which have changes in refractive index relative to neighboring regions (Figure 10) [115]. Additionally, they were able to guide micropipettes toward specific neurons inside live tissue, demonstrating its applicability to assist in microsurgeries. They further used THG and TPEF microscopy to quantitatively assess glioma infiltration in human brain samples [116], where this addressed a well-defined problem in distinguishing normal brain cells from tumors during glioma surgery. Specifically, they were able to assess differences in cellularity and cell morphology when comparing cells from the main tumor mass to those spreading into normal tissue, clearly showing substantial morphological differences in unique glioma types. Moreover, THG images agreed with PET, MRI, and H&E images demonstrating its potential to be used as a clinical auxiliary tool for detecting abnormal areas.

Sun and coworkers further used THG microscopy to explore the neuropathology of fresh, *ex vivo* normal brain tissues from mice and obtained signal from the axons and dendrites from the neocortex, axon fiber bundles from the corpus callosum, and the hippocampus and axonal fiber bundles in the striatum [117]. Importantly, they clearly identified amyloid-*β* plaques (a neuropathological hallmark of Alzheimer’s disease) by performing a correlation with immunohistochemical staining.

## 7. SHG Microendoscopy

As discussed thus far, SHG and THG are powerful tools to examine and probe ECM and cellular organization in the tissue microenvironment in a number of disorders. These studies have largely been performed ex vivo, where in vivo applications have been limited to transparent specimens (e.g., embryos) or at superficial imaging depths (e.g., skin). However, emerging endomicroscopy technology is showing great promise for translating these nonlinear imaging applications from the laboratory benchtop to clinical bedside. Here, we highlight two seminal examples using proximal and distal scanning methods relative to the fiber optic.

**Figure 11:**
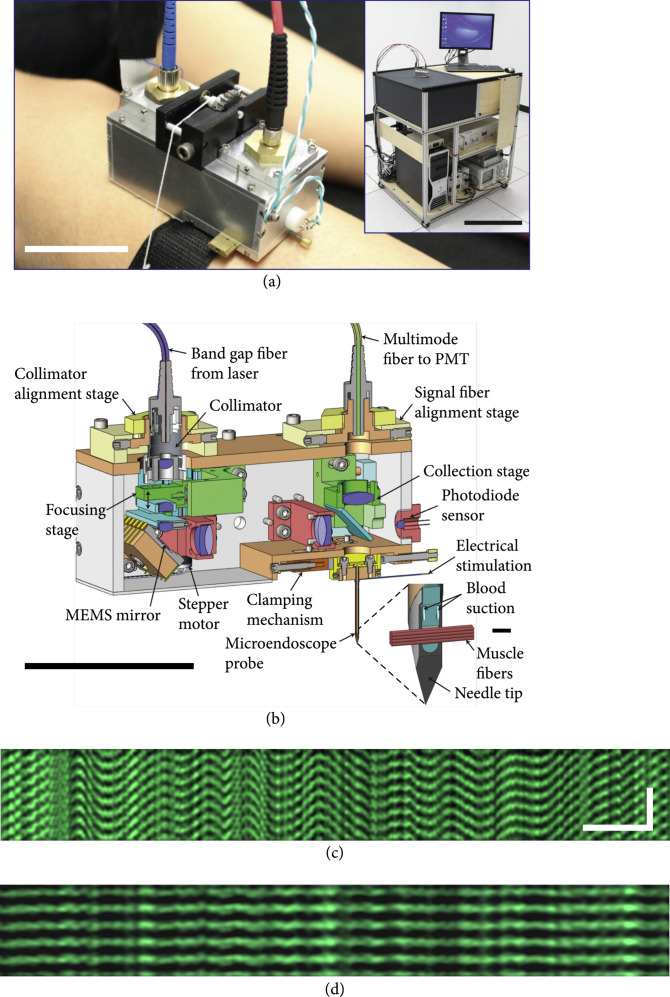
Wearable microendoscope. (a) Demonstration of wearable SHG microscope. The mobile cart houses the light source, photodetector, and computer. (b) CAD rendering of SHG microscope. (c) Line-scanning image of involuntary sarcomere contraction in the relaxed, affected limb of a poststroke subject. (d) Line-scanning image in the unaffected limb of the same subject shown in (c). Reproduced from Ref. [119] and adapted from Ref. [120] with permission.

In a major advance, Schnitzer et al. developed a wearable SHG microendoscope coupled to a laser and scanning electronics via fiber optics [118]. Here, the light is delivered into the muscle via an insertable needle containing a GRIN lens and a stimulating electrode (Figures 11(a) and 11(b)). Using this miniaturized approach, they observed sarcomere lengths and contractile dynamics in human subjects, allowing them to monitor individual motor unit contractions after electrical twitch stimulation. Specifically, they were able to measure and compare sarcomere lengths across six different muscles with ~30 nm precision. For example, they found that unitary contractions in the soleus had slower twitch times than the vastus lateralis, in good agreement with physiology analysis. Additionally, their device captured involuntary contractions and fasciculations and showed significant differences in sarcomere lengths comparing the spastic and contralateral arms in poststroke patients (Figures 11(c) and 11(d)).

**Figure 12:**
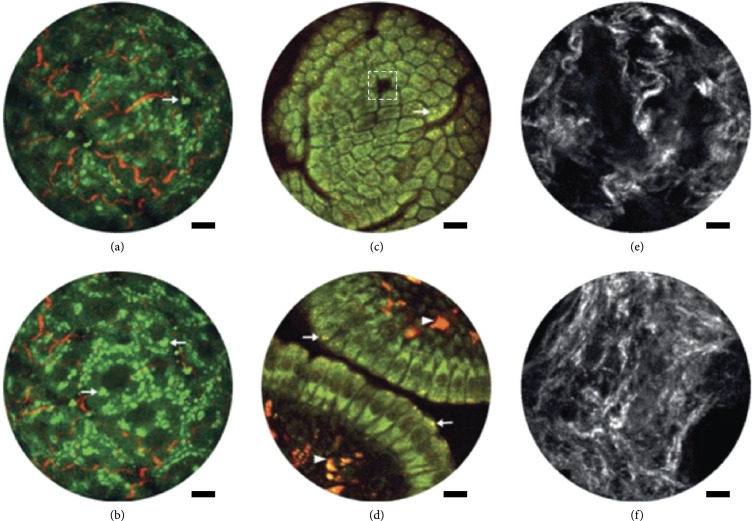
Multimodal nonlinear microendoscope. (a, b) TPEF (green) and SHG (red) images from *ex vivo* mouse liver. (c, d) *In vivo* TPEF (NADH—green and FAD—red) images of the mucosa of mouse small intestine. SHG images of cervical fiber network at gestation day 15 from (e) preterm-birth mouse models and (f) normal pregnant mice. Reproduced from Ref. [121] published under CC BY-NC-ND 4.0.

Li and researchers have developed a distal scanning approach suitable for imaging cavities, where the light is scanned at the exit of the fiber optic. They demonstrated that their SHG/TPEF microendoscope is capable of real-time *in vivo* histological and functional imaging of fresh biological samples [119]. As shown in Figure 12, they found large changes in collagen fiber architecture in the cervix of preterm birth and normal pregnant mouse models, where the latter had a more porous network, consistent with prior benchtop studies [21]. Additionally, they assessed metabolic changes in a mouse kidney ischemia-reperfusion model *in vivo*, which were reversible upon reperfusion.

The key to their more recent successes was the utilization of double clad fibers, corrections of spherical aberrations, and the development of a grating-based dual-fiber for spectrotemporal dispersion compensation [121]. Collectively, these efforts improved the signal collection efficiency by a couple orders of magnitude.

## 8. Perspective and Outlook

Considerable improvements over the last decade in technology and analysis tools have been instrumental in establishing SHG and THG (as individual modalities and also in conjunction with each other and other techniques) as practical optical imaging tools for studying the tissue microenvironment. Nevertheless, many of the techniques discussed here have unique requirements in terms of equipment. For instance, polarization-resolved and SHG/THG physics approaches heavily rely on complex instrumentation and advanced optics to acquire images. Prior to incorporation into nonspecialty microscopy labs and the clinic, many of the concerns related to adding polarization optics and miniaturizing the bulky microscopes and lasers currently used must be addressed. However, small footprint fixed wavelength lasers with sufficient power are already commercially available and more have been recently reported. For example, using a fiber laser-induced super continuum source, Boppart and colleagues have developed a slide-free, compact, and reliable virtual histochemistry tool based on multiphoton microscopy. This technology can simultaneously collect up to four histochemical contrasts, including SHG, THG, TPEF, and three-photon excited fluorescence, in real time [122]. In addition to their enhanced efficiency and miniaturization, fiber optic techniques have advanced, permitting polarization control and analysis.

In contrast, image processing methods can already be easily integrated into both biology labs and clinical settings as they only need a computer with a graphics processing unit for rapid computation. Importantly, recent advances in machine learning have been demonstrated previously in *ex vivo* studies and could be readily implemented in the clinic (where texture analyses are already used for histology), especially when combined with a SHG/THG slide reader with a small footprint laser. This would enable basic researchers and hospitals to build diverse image libraries containing images, from specimens/patients of different sexes, ages, and ethnicities with different genetic information. Additionally, collecting multimodal images in conjunction with deep learning methods would further increase accuracies by offering unique yet complimentary metrics to potentially aid physicians in early diagnosis and monitoring disease progression.

While there have been significant advances in microendoscope technology, several limitations still remain, with the most significant being the achievable depth of penetration. This ultimately limits these devices to rely exclusively on backward detected SHG, which is always lower in intensity than the dominant forward channel. Still, current setups may prove successful in tissue imaging *in vivo* by backward SHG, where for example, this could be done with reversible optical clearing agents, where this has already been demonstrated [123]. Moreover, the backward channel can provide structural information on small, random structures not present in the forward direction. Indeed, we showed this effect in comparing collagen architecture in normal and decorin knockout murine prostate, where the latter results in smaller, disorganized fibrils [49]. While microendoscopes intrinsically have smaller fields of view and numerical aperture in comparison to benchtop setups, their resolution and collection efficiencies have increased tremendously [121]. Thus, they hold great promise for the future.

Lastly, we note that although conventional SHG microscopy collects image stacks through sequential *en face* optical axial sections, this is not a truly 3-dimensional modality. This is because SHG contrast is electric dipole forbidden when fibers are aligned in the direction of laser propagation, and analogously, fibers with high tilt angles have lower SHG intensities. As a result, valuable structural information can be missed. To solve this problem, we have begun to implement SHG microscopy as a tomographic modality to achieve complete 3D imaging [124]. Here, we perform conventional galvo scanning and then cylindrically rotate a specimen, thereby collecting images from multiple excitation angles. We showed by morphological and spatial frequency analysis of images from mouse tail tendon that this approach recovers the 3D structural information. The key to further establishing this approach is optimization of the registration/reconstruction algorithms. We anticipate this becoming a widely used approach for SHG imaging of thick tissues.

Given the increased practicality of SHG and THG microscopies, these modalities have significantly contributed a wealth of knowledge pertaining to the tissue microenvironment, enabling the examination and characterization of cells and ECM components in a wide range of normal and diseased states. Many of the methods could be readily applied to those with access to commercial multiphoton microscopes, in both research labs and clinical settings.
